# Inducing optical self-pulsation by electrically tuning graphene on a silicon microring

**DOI:** 10.1515/nanoph-2022-0077

**Published:** 2022-05-02

**Authors:** Marcus Tamura, Hugh Morison, Bhavin J. Shastri

**Affiliations:** Department of Physics, Engineering Physics and Astronomy, Queen’s University, Kingston, Canada

**Keywords:** graphene, opto-electronic, self-pulsation, silicon photonics

## Abstract

A mechanism for self-pulsation in a proposed graphene-on-silicon microring device is studied. The relevant nonlinear effects of two photon absorption, Kerr effect, saturable absorption, free carrier absorption, and dispersion are included in a coupled mode theory framework. We look at the electrical tunability of absorption and the Kerr effect in graphene. We show that the microring can switch from a stable rest state to a self-pulsation state by electrically tuning the graphene under constant illumination. This switching is indicative of a supercritical Hopf bifurcation since the frequency of the pulses is approximately constant at 7 GHz and the amplitudes initial grow with increasing Fermi level. The CMOS compatibility of graphene and the opto-electronic mechanism allows this to device to be fairly easily integrated with other silicon photonic devices.

## 1 Introduction

Self-sustained pulsations or self-pulsations occur when there is a repetitive firing to a strong stimulus [1]. Self-pulsation has applications in spectroscopy and optical computing [2, 3]. A variety of integrated devices has this behavior and tends to fall into two categories: semiconductor lasers or nonlinear optical cavities. Semiconductor lasers can be classified as being either optically injected [4–12] or electrically injected [1, 13], [14], [15], [16], [17]. The reader is referred to [18] for an in-depth review. However, most nonlinear optical cavity devices are all-optical [19, 20] due to the lack of an electrical injection gain element [21–23]. Without the gain element, achieving optical intensities strong enough to induce nonlinear behavior can be difficult. An electrical input has a lot of benefits as it is more easily integrable with other systems. This is particularly important for cascadability in large scale systems, where integrated electrical gain is more easily implemented compared to optical gain. Additionally electrical inputs can easily interface with CMOS electronic systems. Previous work uses thermal and free carrier effects to obtain self-pulsation in a photonic crystal [22] and a microring [20, 21]. However, these devices are limited to MHz speeds because of their reliance on thermal effects. Faster devices typically use the Kerr effect and free carriers which operate at the femtosecond and nanosecond time scale, respectively. These two effects compete against each other due to their opposite signs in silicon, but the free carrier effect can dominate with higher concentrations in smaller rings [24]. The reason free carriers can create self-pulsation is due to a bifurcation. A bifurcation is a sudden qualitative change in the dynamics of a system when a parameter is smoothly changed. When dynamical systems are perturbed, they typically decay to a nearby constant steady state, also known as a stable fixed point. A stable limit cycle describes an area of state space where nearby states limit towards oscillatory behavior. Self-pulsation is fundamentally created by a bifurcation in the field evolution dynamics that switches trajectories between a stable fixed point to limit cycle behavior. A type of bifurcation that can cause this is a supercritical Hopf bifurcation, where by definition a stable fixed point is converted to an unstable fixed point surrounded by limit cycle. Bifurcations require nonlinear behavior, so nonlinear materials could enhance self-pulsation; one such material is graphene.

Graphene comes with a few benefits: Its linear dispersion means it is wavelength independent. It can operate over a large bandwidth because it has no bandgap. It is CMOS compatible [25], which allows for relatively easy integration with other silicon photonic devices. Its absorption tunability is strong since it is related to Pauli blocking. When graphene is electrically charged, its Fermi level increases and fills electron states. Due to the Pauli exclusion principle, photons cannot excite electrons to filled states, so the absorption drops significantly. There are a variety of devices that use this electrical tunability as a modulator [25, 26] at GHz speeds. However, current fabrication technology for graphene-metal contacts can suffer from reproducibility issues which can cause issues for scalability [27]. Most current theories describe graphene as a surface conductivity [28]. Its nonlinear behaviors at high light intensities are captured in intensity dependence of the surface conductivity. Graphene exhibits two major nonlinear effects: saturable absorption and the Kerr Effect. At low optical intensity, the absorption is large until the intensity reaches the saturation intensity. Beyond the saturation intensity the absorption decreases [29]. Saturable absorption is particularly important for nonlinear devices as it can improve the thresholding behavior [18, 30]. The saturation intensity depends primarily on the electron scattering rate and wavelength and does not change with Fermi level. The Kerr effect in graphene is overall less understood. The Kerr coefficient in graphene has many contradictory values in the literature since it strongly depends on the Fermi level, electron scattering rate, frequency, and type of light polarization [31, 32]. There is also evidence that it depends on laser pulse width as well [33] at the femtosecond scale. Theoretically the dependence of the Kerr effect on Fermi level is especially strong near the Dirac point where the conduction and valence bands meet and where graphene is most often measured. This has led to measured Kerr coefficient values orders of magnitude different and occasionally with different sign [34, 35]. However, all seem to agree that the Kerr coefficient in graphene is unusually large. In an integrated device, the mode overlap with graphene will significantly affect how strong the Kerr effect is on the overall mode. In this paper, we propose a graphene on silicon microring device capable of switching between stable and self-pulsation states by electrically tuning the graphene. This device is optoelectronic and CMOS compatible, making it easily integrated with silicon photonic platforms and CMOS electronics. We develop a numerical model, considering all relevant nonlinear effects in silicon and graphene and discuss how the optical nonlinearities of graphene can be electrically tuned. The dynamics of the light energy and free carrier concentration will be investigated through simulation and show a supercritical Hopf bifurcation is responsible for the switch to self-pulsating behavior.

## 2 Device design

**Figure 1:**
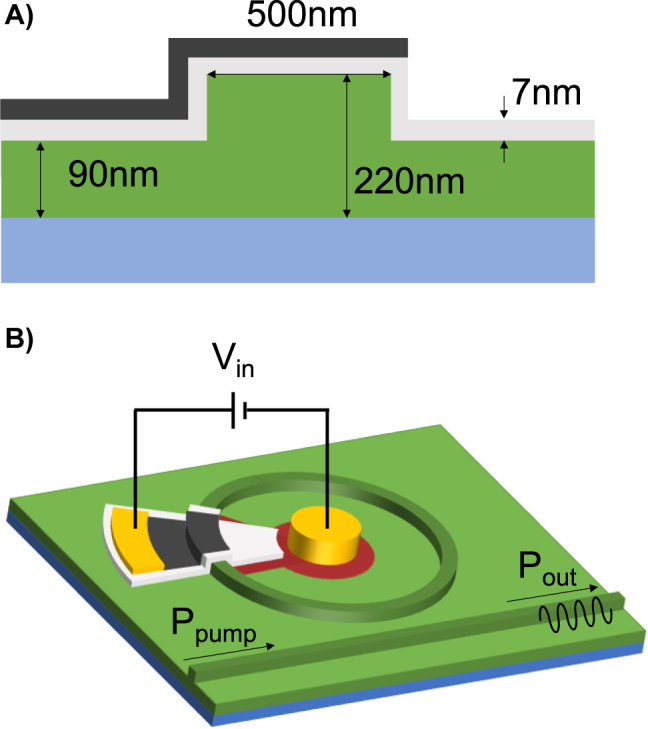
The physical structure of the proposed device. Note the diagram is not to scale for clarity. (A) The cross section where the graphene (black) is over top of the silicon rib waveguide (green) separated by a gate aluminum oxide layer (gray). The waveguide is over silicon dioxide (cyan). (B) The microring resonator with a bus waveguide that carries the input and output power. Voltage bias is applied to metal contacts (gold) and doped silicon (red) to change the Fermi level of the graphene.

The structure of the studied device is given in Figure 1 with the dimensions given in Table 1. We use a silicon microring resonator with a bus rib waveguide on an (silicon-on-insulator) SOI platform. Over the silicon microring, a 7 nm thick Al_2_O_3_ gate layer is deposited. Graphene is placed over the gate oxide over top of the waveguide and covers 10 percent of the ring. Metal contacts are deposited over the graphene and doped silicon. The contacts are sufficiently far away from the waveguide to prevent optical losses. By applying a bias to the graphene across the aluminum oxide, the graphene can be charged and its Fermi level can be modulated. To find the distribution of the electric field in the waveguide a finite difference eigenmode solver was used. The horizontal electric field forms the majority of the energy in the mode. The interaction of the light with the graphene is strongest when the electric field is parallel to the graphene because it is treated as a surface conductivity. We operate the ring in quasi TE-mode and in this way the graphene on top of the waveguide has more influence than the graphene on the side of the waveguide. Applying a voltage bias to the graphene changes its optical properties; however the overall distribution of the light in the waveguide changes very little. We approximate the optical energy distribution in the waveguide to be constant with changing Fermi level of graphene. Voltage bias does change the effective refractive index and these effects are captured in the next section as perturbations. We obtain the unperturbed effective refractive index, *n*_0_, from the eigenmode solver when the Fermi level is 0.1 eV. This Fermi level was chosen because the optical behavior of graphene is relatively constant there.

**Table 1:** Geometrical parameters.

Symbol	Value	Definition
R	5 um	Ring radius
W	500 nm	Rib width
H	220 nm	Rib height
h	90 nm	Slab height
*d*_*Al*_2_O_3__	7 nm	Aluminum oxide thickness

## 3 The numerical model

The optical behavior of the ring is captured by treating it as a cavity [24, 36]. We use the cavity Eq. (1) to simulate the behavior of the ring. The complex amplitude *u* is normalized so that |*u*|^2^ is the energy of the mode in the ring. Light is coupled into the ring via a nearby bus waveguide with input power *P*_pump_. The external quality factor *Q*_*e*_ determines the strength of the coupling Γ_*c*_ = *ω*_0_/*Q*_*e*_. The imaginary terms in (1) correspond to dispersion effects and the real terms correspond to absorption effects. The detuning parameter is *δ* = *ω*_0_ − *ω*_*d*_, where *ω*_0_ is the resonant frequency of the ring and *ω*_*d*_ is the frequency of the input mode. Using the detuning parameter, the ring is biased close to resonance. The cavity itself has an internal quality factor *Q*_0_ which encompasses the constant linear losses (radiation, bending, sidewall roughness, etc.). *Q*_*l*_ is the loaded quality factor, taken to be half of *Q*_0_ to approach critical coupling.

(1) $$\frac{\partial u}{\partial t}=\sqrt{\Gamma_{c}P_{pump}}+u\left(-\frac{\omega_{0}}{2Q_{l}}+i\left(\delta +\Delta \tilde{\omega }\right)\right)$$

The output power of the bus waveguide is described by Eq. (2)

(2) $$P_{out}=|\sqrt{P_{pump}}-\sqrt{\Gamma_{c}}u{|}^{2}$$

The values chosen for the numerical simulation are shown in Table 2. We follow [37] and use first order variation theory to take a weighted average (3) of the local relative change in the complex refractive index over the energy in the cavity (4). The real part of the complex frequency $\Delta \tilde{\omega }$ can be thought of as a perturbation to the resonant frequency of the cavity. The imaginary part corresponds to a perturbation in the rate of light absorption in the ring. The surface conductivity of graphene is complex, so its imaginary part stores a very small amount of the energy in the ring.

(3) $$\Delta \tilde{\omega }=-\frac{\begin{array}{c} \omega_{0}\int_{cavity}\Delta \tilde{\epsilon }\left(r\right)|E\left(r\right){|}^{2}{\text{d}}^{3}r-i\int_{graphene}\tilde{\sigma }|E_{\|}\left(r\right){|}^{2}{\text{d}}^{2}r \end{array}}{4W_{cavity}}$$

(4) $$\begin{array}{ll} W_{cavity} & =\frac{1}{4}\underset{cavity}{\int }\left[\epsilon_{0}\epsilon_{r}\left(r\right)|E\left(r\right){|}^{2}+\mu_{0}|H\left(r\right){|}^{2}\right]{\text{d}}^{3}r\\ & \;+\frac{1}{4}\underset{graphene}{\int }\frac{\partial {\tilde{\sigma }}_{Im}^{\left(1\right)}}{\partial \omega }|E\left(r\right){|}^{2}{\text{d}}^{2}r \end{array}$$

**Table 2:** Simulation parameters.

Symbol	Value	Definition
*Q*_0_	6E4	Intrinsic quality factor
*Q*_e_	6E4	External quality factor
*P*_pump_	25 mW	Input power
*λ*_0_	1550 nm	Resonant wavelength
*δ*	−0.000055*ω*_0_	Frequency detuning
*τ*_car_	100 ps	Free carrier lifetime
*n*_0_	2.265479	Unperturbed effective refractive index
*β*_2Si_	7.5E − 12 m/W	TPA coefficient for silicon
*n*_2Si_	4.5E − 18 m^2^/W	Kerr coefficient for silicon
Γ	10 meV	Graphene relaxation rate
*W*_sat_	0.14 pJ	Saturation cavity energy
*σ*_FCA_	1.45E − 21 m^2^	Free carrier absorption coefficient
*σ*_e_	8.8E − 28 m^3^	Electron dispersion coefficient
*σ*_h_	1.35E − 22 m^3^	Hole dispersion coefficient

We can account for the free carrier and the nonlinear bulk effects on the dispersion and absorption in the cavity by using the first term in Eq. (3). Since the change in refractive index is small compared to the refractive index of silicon we can use (5).

(5) $$\Delta \epsilon_{r}=2\Delta nn+{\left(\Delta n\right)}^{2}\approx 2\Delta nn$$

The effect of free carriers on refractive index and absorption are well known [38] and are captured in the *σ*_FCA_, *σ*_e_, and *σ*_h_ terms in (6). Like [39], we assume that the free carrier concentration, *N*, in the waveguide is roughly uniform due to diffusion effects and can be pulled out of the integral. We only account for the free carriers in the rib of the waveguide so that *V*_car_ = 2*πRwH* instead of integrating over the whole microring cavity which includes the graphene, oxides and surrounding air.

(6) $$\Delta \omega_{\text{FC}}=\omega_{0}\gamma_{\text{FC}}\left(\sigma_{\text{e}}N+\sigma_{\text{e}}N^{0.8}+i\sigma_{FCA}\frac{c}{2\omega_{0}}N\right)$$

The dimensionless constant *γ*_FC_ encapsulates the weighted average over the mode energy.

(7) $$\gamma_{\text{FC}}=\frac{\underset{V_{car}}{\int }n_{\text{Si}}|E\left(r\right){|}^{2}\text{d}r}{2W_{cavity}}$$

A similar procedure is carried out for two-photon absorption and the Kerr effect in silicon. However, the averaging over the mode energy is presented as an effective nonlinear volume as is historically common [40]. This is shown in (8) and (9) respectively. Note that we only consider two photon absorption in the silicon, since the effect is negligible in the oxides and in graphene. The Kerr effect in graphene comes in later using the surface conductivity model.

(8) $$V_{TPA}=\frac{\beta_{2}}{n_{0}^{2}}\frac{{\left(2W_{cavity}\right)}^{2}}{\epsilon_{0}^{2}\underset{silicon}{\int }n_{\text{Si}}^{2}\beta_{2Si}|E\left(r\right){|}^{4}\text{d}r}$$

(9) $$V_{Kerr}=\frac{n_{2}}{n_{0}^{2}}\frac{{\left(2W_{cavity}\right)}^{2}}{\epsilon_{0}^{2}\underset{silicon}{\int }n_{\text{Si}}^{2}n_{2Si}|E\left(r\right){|}^{4}\text{d}r}$$

The optical behavior in graphene is governed by its conductivity. The linear conductivity is well-known and is given as (10) and (11), the interband and intraband contributions, respectively.

(10) $$\sigma_{interband1}=\frac{-ie^{2}}{4\pi \hbar }\ln\frac{2|E_{\text{f}}|-\hbar \omega_{0}+2i\Gamma }{2|E_{\text{f}}|+\hbar \omega_{0}-2i\Gamma }$$

(11) $$\begin{array}{ll} \sigma_{intraband1} & =\frac{-i{\text{e}}^{2}k_{\text{B}}T}{\pi \left(\hbar^{2}\omega_{0}^{2}-2i\Gamma^{2}\right)}\\ & \;\times \left(\frac{E_{f}}{k_{\text{B}}T}+2\;\ln\left(1+{\text{e}}^{-E_{f}/k_{\text{B}}T}\right)\right) \end{array}$$

**Figure 2:**
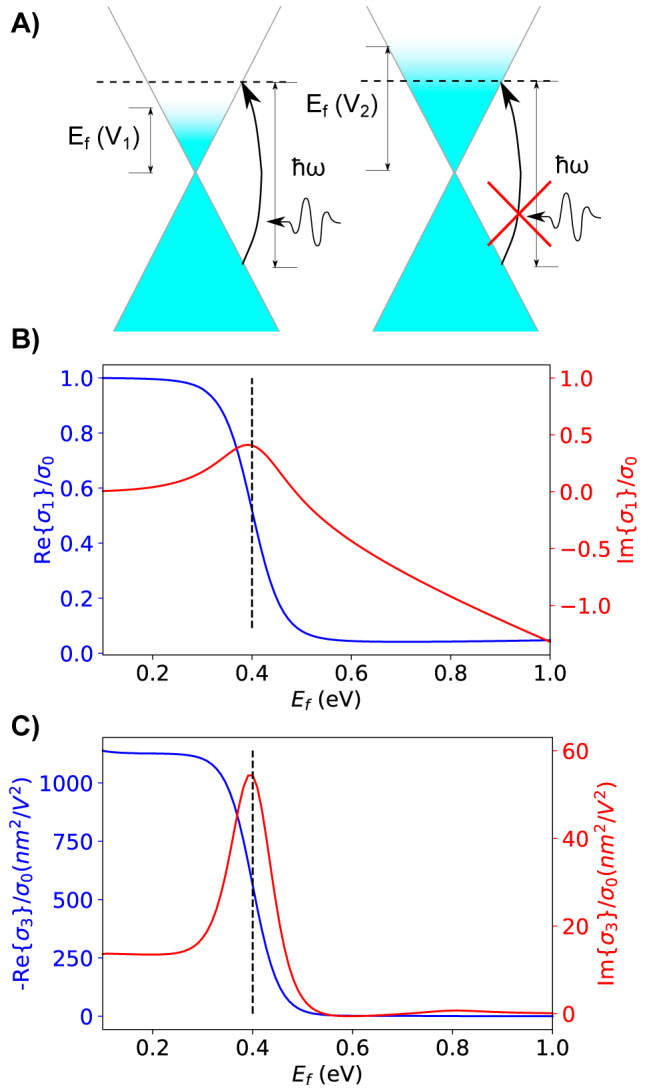
The effect of Fermi level on the conductivity of graphene. (A) Pauli blocking in graphene. The Fermi level allows or prevents photon absorption in graphene and is tuned by applying voltage |*V*_1_| < |*V*_2_|. (B) Linear surface conductivity of graphene as a function of Fermi level. (C) Third order surface conductivity of graphene as a function of Fermi level. Both (B) and (C) assume *λ*_0_ = 1550 nm, Γ = 10 meV, *T* = 300*K* and are normalized by *σ*_0_ = *e* ^2^/(4*ℏ*).

Only the intraband contribution can account for the thermal distribution of electrons around the Fermi level analytically, so numerical integration with (12) must be used to capture these thermal effects for the interband contribution. Note this formula also applies for the third order conductivity. The thermal distribution softens the threshold for Pauli blocking which is the main contributor to the Fermi level dependence (Figure 2A). All results presented assume 300 K.

(12) $$\sigma_{1,3}\left(E_{\text{f}},T\right)=\overset{\infty }{\underset{-\infty }{\int }}\;\text{d}E\frac{1}{1+{\text{e}}^{\left(E-E_{\text{f}}\right)/k_{\text{B}}T}}\frac{\partial }{\partial E}\sigma_{1,3}\left(E,0\right)$$

Using the second term of (3) on the complex surface conductivity of graphene, we arrive at (13). We purposefully look at the change in the imaginary conductivity relative to 0.1 eV so that the ring is on resonance when *ω*_d_ = *ω*_0_ in low light intensity conditions. The first term will saturate at high intensities representing saturable absorption of the graphene.

(13) $$\begin{array}{ll} \Delta {\tilde{\omega }}_{\text{gr}} & =i\frac{\underset{graphene}{\int }\sigma_{Re}^{\left(1\right)}\left(E_{f}\right)}{\sqrt{1+\frac{3|u{|}^{2}}{W_{sat}}}4W_{cavity}}\\ & \;-\frac{\underset{graphene}{\int }\left(\sigma_{Im}^{\left(1\right)}\left(E_{f}\right)-\sigma_{Im}^{\left(1\right)}\left(0.1\;eV\right)\right)|E_{\|}\left(r\right){|}^{2}{\text{d}}^{2}r}{4W_{cavity}} \end{array}$$

Saturable absorption has been modelled nonperturbatively as it gives more accurate values when the intensity *I* is above the saturation intensity *I*_sat_. It has been shown that the absorption of graphene decreases by $1/\sqrt{I}$ due to its linear dispersion, unlike many other materials with saturable absorption which decrease by 1/*I* [29]. Note that like others, we assume that the light in the graphene is approximately uniform to eliminate the saturable absorption effect on the weighted average over the whole waveguide [23, 36]. However, this approximation breaks down when comparing the graphene on top of the waveguide with that on the left wall of the waveguide. This is because the graphene only affects the tangential field which is small on the left wall. Inherently, this means the top graphene saturates before the left graphene, and would effectively cause saturation effects at two different cavity energies. This effect is small overall and can be safely ignored when just the top graphene is counted. Like [23, 36] we find the saturation cavity energy using Eq. (14), however in our case it is un-normalized.

(14) $$W_{sat}=\frac{2I_{sat}W_{cavity}}{c\epsilon_{0}|E_{\|}{|}^{2}}$$

Graphene as a material has a significant Kerr coefficient. The Kerr effect is strongly dependent on the Fermi level and comes from the third order surface conductivity. Since we are only interested in the Kerr effect, and not four-wave mixing we refer to *σ*_3_(*ω*, *ω*, − *ω*) as simply *σ*_3_. The expression of the third order conductivity of graphene is long and complicated, but is given in [34] and is graphically shown Figure 2C. We use this and the application of first order perturbation theory given in [37] to obtain (15)

(15) $$\Delta \omega_{grKerr}=-3\frac{\underset{graphene}{\int }\sigma_{Im}^{\left(3\right)}\left(E_{f}\right)|E_{\|}\left(r\right){|}^{4}{\text{d}}^{2}r}{16W_{cavity}^{2}}|u{|}^{2}$$

We restate Eq. (1), with all the perturbations accounted for in equation (16). This equation has a number of implicit assumptions, such as assuming the distribution of light energy in the mode changes very little with changing the Fermi level and the electric field amplitude is roughly uniform over the graphene. The change in the refractive index in silicon is small compared to its unperturbed refractive index.

(16) ∂u∂t=ΓcPpump+u−ω02Ql+σFCAcNγFC2 +β2c22n02|u|2VTPA+Im(Δω~gr)+iδ+ω0γFCσeN+σhN0.8−n2cω0VKerrn02|u|2 +Re(Δω~gr)+ΔωgrKerr

Equation (16) describes the generation of free carriers by two photon absorption and decay according to an characteristic lifetime *τ*_car_.

(17) $$\frac{\partial N}{\partial t}=\frac{c^{2}\beta_{2}}{n_{0}^{2}2\hbar \omega_{0}V_{TPA}V_{car}}|u{|}^{4}-\frac{N}{\tau_{car}}$$

The interplay in the dynamics between equations (16) and (17) is which leads to the self-pulsation behavior.

## 4 Results and discussion

Due to the Pauli exclusion principle, it is Fermi level relative to half the photon energy that primarily determines conductivity. If the Fermi level is below half the photon energy, an electron can be excited from the valence band to the conduction band. However, if the Fermi level is above half the photon energy, an electron cannot be excited because the state in the conduction band is filled. In this way, the optical behavior of graphene is switched on and off by changing the Fermi level. The temperature and electron scattering rates effectively blur the states between on and off by introducing a probability whether the state is filled or not. They primarily only have an effect when the Fermi level is roughly equal to half the photon energy. The change in absorption is the dominant effect overall, but the dispersion effects also change. The change in the effective refractive index of the ring is minor due to the small portion of light in the graphene compared to the rest of the waveguide. Graphene as a material has a strong Kerr effect, however in this device its overall effect is negligible compared to the Kerr effect in silicon. This is primarily because there is far more silicon in the microring compared to graphene. Additionally, the electric field is stronger in the middle of the waveguide and weaker at the surface where graphene sits. The absorption of graphene is still the dominant effect, and dominates over the linear and two-photon absorption loss in the ring when it is on. This is important, because it controls the overall photon lifetime in the ring. In order for self-pulsation to occur, the photon lifetime and the free carrier lifetime must be roughly the same order of magnitude. When they are not the same order of magnitude, the ring decays to a constant steady state.

**Figure 3:**
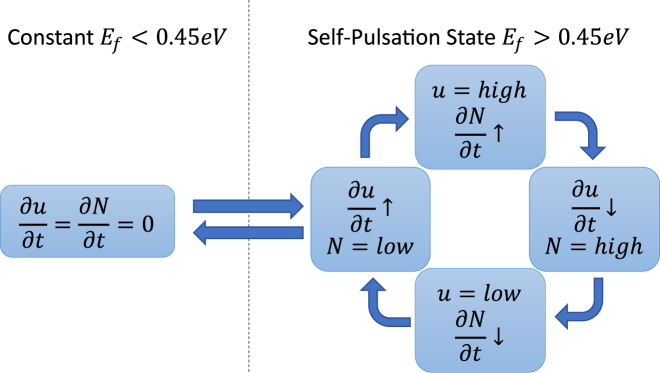
Flow chart of the self-pulsation behavior. The dotted line indicates the bifurcation between the constant state and the self-pulsation state.

The self-pulsation behavior comes from the oscillations of the free carrier concentration. The nonlinear behavior of the graphene has very little to do with the self-pulsation. In fact, the saturable absorption makes switching more difficult at higher light intensities because the change in absorption is smaller. The electrical tunability of the linear absorption of graphene merely controls the photon lifetime in the ring. When the Fermi level is low, the absorption is high, and light is absorbed before it has the chance to generate a significant number of free carriers. When the Fermi level is high, the absorption is low, and light is allowed to generate free carriers. The free carriers begin to dominate the absorption and dispersion effects. As free carriers increase, the absorption increases and the ring is pushed further away from resonance (see Figure 3). This causes less light to build up in the ring, so the free carriers decrease. With less free carriers, the absorption decreases and the ring is pushed towards resonance causing light build up again. Thus free carriers increase and the cycle begins again. Two photon absorption and the Kerr effect are both present during these cycles as well, but they are smaller overall. Generally speaking, two photon absorption has less of an effect than the absorption of the free carriers it induces. The Kerr effect typically competes with the free carriers in silicon based devices due to their opposite signs. The Kerr effect in graphene slightly hinders the silicon Kerr effect with its negative Kerr coefficient; however this change in the total Kerr effect in the ring is negligible because the mode overlap is significantly higher over the silicon than the graphene. This is especially true when the Fermi level is above 0.5 eV when the imaginary third order conductivity becomes a small value.

**Figure 4:**
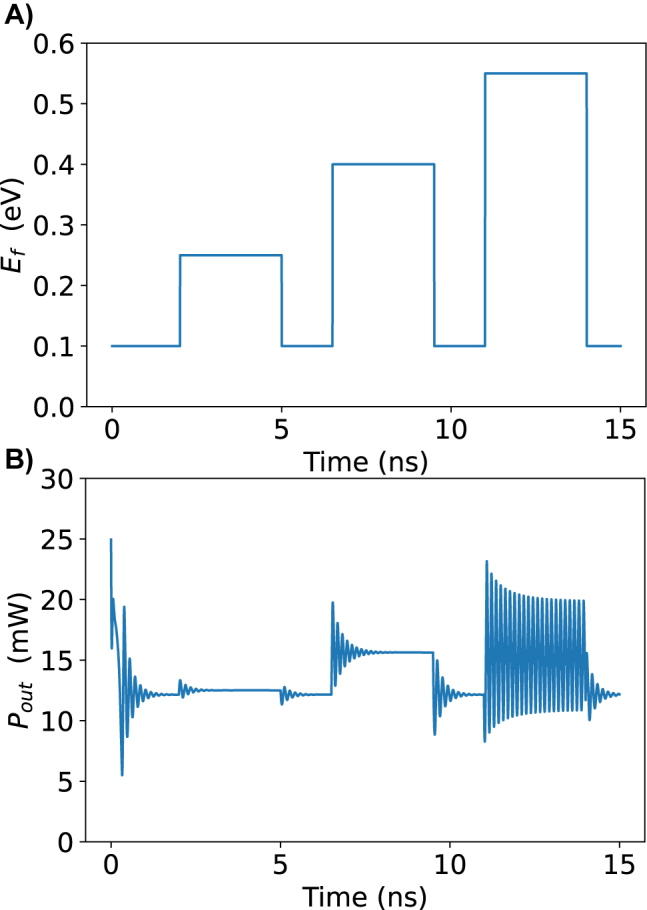
Tuning the Fermi level to induce dynamic behavior. (A) The applied Fermi level pulsed in time. (B) The resultant output power in the bus waveguide.

**Figure 5:**
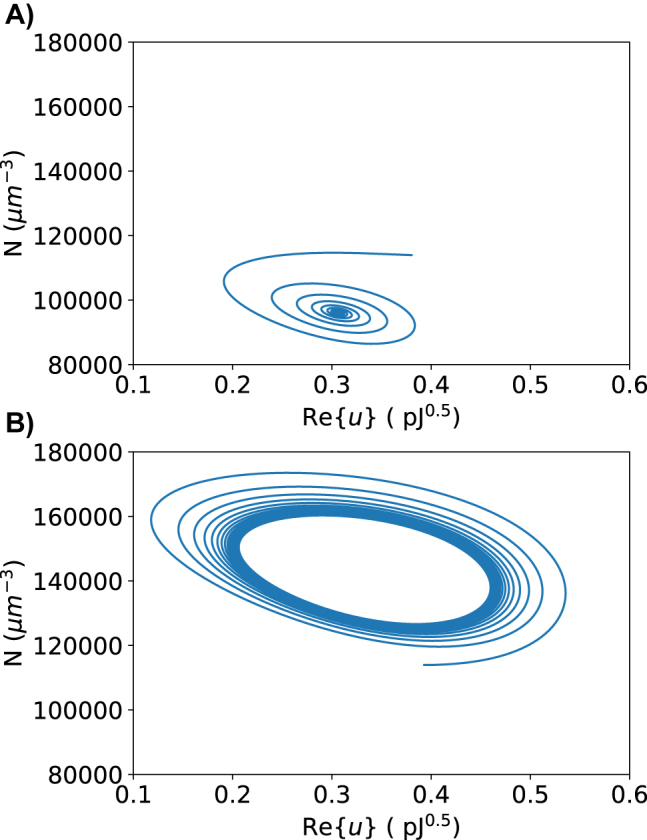
Phase diagram before and after the supercritical Hopf bifurcation. (A) Fermi level is set to 0.4 eV. Oscillations decay to a stable fixed point. (B) Fermi level is set to 0.55 eV. Oscillations are sustained in a stable limit cycle.

**Figure 6:**
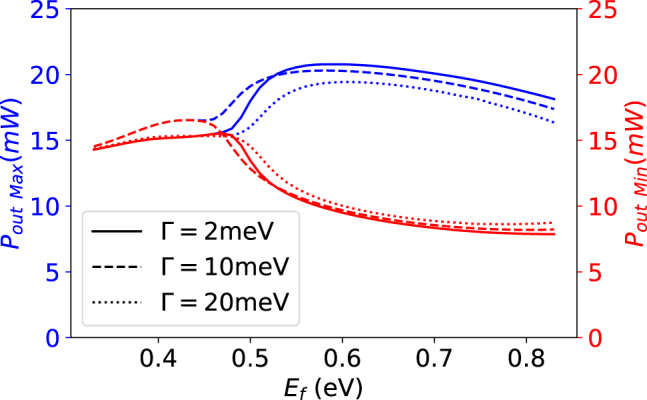
The maxima and minima of the output power after the transient effects have subsided. Before the bifurcation, the system has a stable fixed point so the maximum and minimum output power is identical. After the bifurcation self-pulsation begins so the maximum and minimum separate and the amplitudes grow. The relaxation rate of graphene has a small effect on the exact position of the bifurcation.

**Figure 7:**
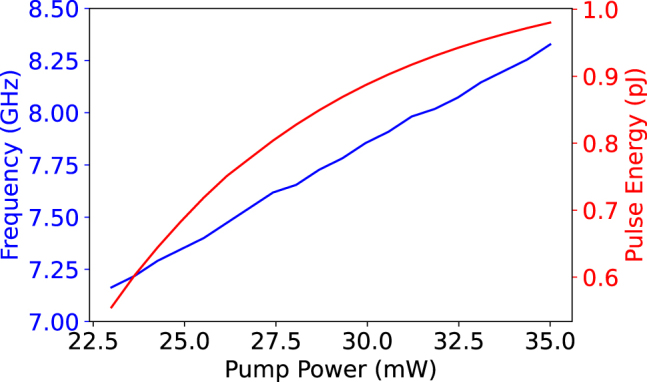
The pulsation frequency and pulse energy as the pump power is varied. *E*_f_ = 0.6 eV and Γ = 10 meV.

Figure 4 shows some important behavior for the system. The first pulse does not change the output power by much because the Fermi level is still far enough away from half the photon energy that Pauli blocking does not take place. The second pulse brings the Fermi level equal to half the photon energy, so Pauli blocking occurs, but not completely due to scattering and temperature effects around the Fermi level. This is enough to perturb the system off of its previous fixed point. The system oscillates a bit until it settles on the new fixed point. Note that the fixed point is still stable, a bifurcation has not occurred yet. When the second pulse ends, there is an oscillatory decay back to the original fixed point. The large third pulse is enough to cause the bifurcation. Previously, the oscillations settled to a fixed point, but now that fixed point has become unstable and trajectories progress away from the point and form a stable limit cycle around it. This is shown as the oscillations reach a constant height and self-pulsation continues until it is turned off by lowering the Fermi level again. The dynamics of the second and third pulse are shown in Figure 5 as phase space diagrams. The amplitude of the oscillations can change with the input Fermi level Figure 6. At 0.45 eV, the output power barely begins oscillating and the maximum and minimum of the peaks increasing. The frequency of pulses is approximately 7.35 GHz when the input power is 25 mW. The frequency of the system can change slightly with pump power (see Figure 7). The frequency increase is due to the increase in the generation of free carriers. The frequency can also be designed to slightly different values by using a microring ring with a different quality factor and free carrier lifetime. Note this system displays no hysteresis; the oscillations can be turned on and off by returning the Fermi level back to its original state. The frequency invariance and growth of the oscillations with the bifurcation parameter indicate that this is a supercritical Hopf bifurcation.

## 5 Conclusions

We have discussed the electrical tunability of the linear and nonlinear optical effects in graphene. This paper has described how graphene on a silicon microring can be modelled in coupled mode theory. The optical properties of graphene were shown to change the behavior of the microring and were electrically tunable. We have demonstrated that this tunability can be utilized to switch the microring from stable state to a self-pulsating state. This effect is fundamentally due to Pauli blocking and disappearance of the interband conductivity at high Fermi energies. The self-pulsation behavior is due to the oscillations of the free carrier concentration in the microring. The frequency of the pulses was roughly 7 GHz and could be slightly tuned by adjusting the pump power. This device used graphene primarily for its electrically tunable absorption to effectively switch from a low-intensity linear regime to a high intensity nonlinear regime. In principle, this could be used in other devices to electrically activate other nonlinear behaviors.
